# RvE1 Impacts the Gingival Inflammatory Infiltrate by Inhibiting the T Cell Response in Experimental Periodontitis

**DOI:** 10.3389/fimmu.2021.664756

**Published:** 2021-05-03

**Authors:** Carla Alvarez, Henrique Abdalla, Salwa Suliman, Paola Rojas, Yu-Chiao Wu, Rawan Almarhoumi, Ren-Yeong Huang, Mario Galindo, Rolando Vernal, Alpdogan Kantarci

**Affiliations:** ^1^ Forsyth Institute, Cambridge, MA, United States; ^2^ Laboratory of Neuroimmune Interface of Pain Research, Faculdade São Leopoldo Mandic, Instituto de Pesquisas São Leopoldo Mandic, Campinas, Brazil; ^3^ Department of Clinical Dentistry, Center for Clinical Dental Research, University of Bergen, Bergen, Norway; ^4^ Harvard School of Dental Medicine, Boston, MA, United States; ^5^ School of Dentistry, National Defense Medical Center, Taipei, Taiwan; ^6^ Program of Cellular and Molecular Biology, Institute of Biomedical Sciences (ICBM), Faculty of Medicine, Universidad de Chile, Santiago, Chile; ^7^ Millennium Institute on Immunology and Immunotherapy, Faculty of Medicine, University of Chile, Santiago, Chile; ^8^ Periodontal Biology Laboratory, Dentistry Faculty, Universidad de Chile, Santiago, Chile

**Keywords:** T cells, Periodontal disease, inflammation, RvE1, SPMs (specialized pro-resolving mediators)

## Abstract

Periodontitis is a chronic inflammatory disease associated with the formation of dysbiotic plaque biofilms and characterized by the progressive destruction of the alveolar bone. The transition from health to disease is characterized by a shift in periodontal immune cell composition, from mostly innate (neutrophils) to adaptive (T lymphocytes) immune responses. Resolvin E1 (RvE1) is a specialized pro-resolution mediator (SPMs), produced in response to inflammation, to enhance its resolution. Previous studies have indicated the therapeutic potential of RvE1 in periodontal disease; however, the impact of RvE1 in the microbial-elicited osteoclastogenic immune response remains uncharacterized *in vivo*. In the present study, we studied the impact of RvE1 on the gingival inflammatory infiltrate formation during periodontitis in a mouse model. First, we characterized the temporal-dependent changes of the main immune cells infiltrating the gingiva by flow cytometry. Then, we evaluated the impact of early or delayed RvE1 administration on the gingival immune infiltration and cervical lymph nodes composition. We observed a consistent inhibitory outcome on T cells -particularly effector T cells- and a protective effect on regulatory T cells (Tregs). Our data further demonstrated the wide range of actions of RvE1, its preventive role in the establishment of the adaptive immune response during inflammation, and bone protective capacity.

## Introduction

Periodontitis is a chronic inflammatory disease characterized by the progressive destruction of the tooth‐supporting apparatus, comprised of the periodontal ligament, radicular cement, and alveolar bone (1, 2). While the microorganisms that colonize the dental biofilm are considered as primary etiological agents, the main determinant of the disease progression is the host’s immune response against the microbial challenge (3). Due to a chronic stimulation by the continuous presence of microorganisms, various types of immune cells infiltrate the periodontal tissues under physiological conditions, keeping a state of mild inflammation (4). Among these patrolling cells, neutrophils are significantly enriched and play a critical role in periodontal homeostasis while the lymphocytic compartment, particularly CD3^+^ T cells, is the dominant population with the vast majority bearing a CD45RO^+^ memory phenotype (4).

The transition from health to disease in periodontal tissues is characterized by a shift in immune cell composition. There is an increase in the proportion of neutrophils (CD15^+^CD16^+^ cells), which may possess a hyper-inflammatory phenotype characterized by the over-expression of reactive oxygen species and proinflammatory cytokines (IL-1β, IL-6, IL-8, and TNF-α) (4, 5). Tissue-resident macrophages are expanded, and circulating monocytes are recruited to be differentiated into macrophage-like cells (6, 7). The chronic inflammatory infiltrate in periodontal tissues during disease is mostly constituted by CD3^+^ T cells (4, 8). CD4^+^ Th17 cells are significantly expanded by the microbial dysbiosis around the tooth, requiring local IL-6 and IL-23 production for their expansion (8, 9). Th17 cells are key drivers of the host defense against a dysbiotic microbial community, as demonstrated by the inhibition of Th17 cells accumulation in the oral mucosa and draining lymph nodes due to broad-spectrum antibiotic treatment in the periodontitis model (9). These cells are also critical players in the induction of bone loss by directly expressing RANKL and being the primary producers of IL-17, which further promotes RANKL expression by periodontal ligament fibroblasts and osteoblasts (9). These observations suggest a critical role for T cell-mediated transition from health to periodontitis and an osteoimmunological basis for destruction of alveolar bone (3). It is unclear whether this process can be reversed by restoring the inflammatory and immunological homeostasis in periodontal tissues.

Unlike the anti-inflammatory process where inflammation is blocked, resolution of inflammation is an active phenomenon regulated by specific mediators named specialized pro-resolution mediators (SPMs), a genus of endogenous lipid mediators that include separate molecular families, such as lipoxins, resolvins, protectins, and maresins (10). Resolvin E1 (RvE1, 5S,12R,18R-trihydroxy-6Z,8E,10E,14Z,16E-EPA) is an SPM produced by converting eicosapentaenoic acid (EPA) into 18R-hydro(peroxy)-eicosapentaenoic acid (HEPA), which is metabolized by activated leukocytes into RvE1 (11). RvE1 is produced in response to the activation of inflammation, where it enhances the resolution phase by decreasing neutrophil chemotaxis and enhancing a non-phlogistic and macrophage-directed clearance of apoptotic neutrophils (12). Evidence over the years has further highlighted the wide range of actions of RvE1 on different cell types, such as inhibiting osteoclast differentiation and bone resorption capacity (13), attenuates the damage of macrophages by induced oxidative stress (14), inhibits excessive proliferation of fibroblasts after injury (15), regulates platelet activation (16), inhibits dendritic cell migration (DCs), and T cell activation (17).

RvE1 prevents and reverses the alveolar bone loss in experimental periodontitis in rats and rabbits (18–21). *In vitro* studies of RvE1’s effect on neutrophils from patients with periodontitis showed that it reduced their superoxide production (18). The treatment with RvE1 enhanced the phagocytic capacity of macrophages from patients with aggressive periodontitis to a similar level to that of healthy controls, indicating the possible rescue of the phagocytic activity (16). Even though RvE1 is not intrinsically antibacterial, the prevention and treatment of periodontitis with topical RvE1 markedly modulated the oral microbial composition in a rat model of periodontitis (20). The impact of RvE1 in the microbial-elicited osteoclastogenic immune response remains uncharacterized *in vivo*. In the present study, we sought to investigate the impact of RvE1 on the gingival inflammatory infiltrate formation during periodontitis in a mouse model. We characterized the temporal-dependent changes of the main immune cells infiltrating the gingiva and the impact of RvE1 administration. We observed a consistent inhibitory outcome on T cells -particularly effector T cells- and a protective effect on regulatory T cells (Tregs). Our data further demonstrated the wide range of actions of RvE1, its preventive role in the establishment of the adaptive immune response during inflammation, and bone protective capacity.

## Results

### Characterization of the Gingival Inflammatory Infiltrate During Periodontitis

**Figure 1:**
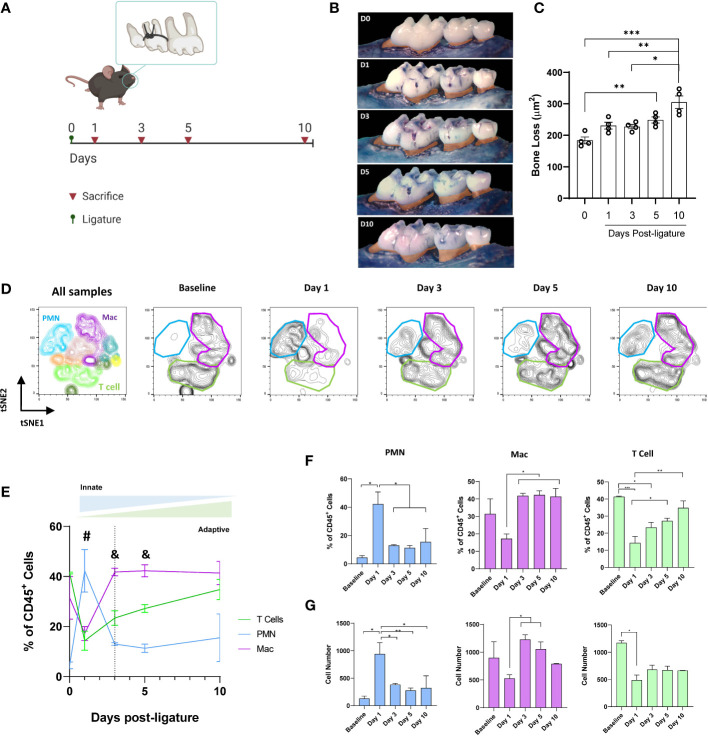
Gingival immune-infiltrate shift during experimental periodontitis progression. **(A)** Experimental design. **(B)** Representative palatal views of maxillary molars from animals at baseline (D0) or sacrificed at days 1, 3, 5, or 10 after ligature placement. Bone loss area is highlighted in orange. **(C)** Bone loss measurements from the cementoenamel junction to alveolar bone crest. **(D)** Identification of gingival neutrophils (PMN, CD45^+^LY6G^high^ LY6C^mid^CD11b^+^), macrophages (Mac, CD45^+^CD64^+^CD11b^+^MHCII^+^), and T cells (CD45^+^CD3^+^) as clustered by FlowSOM. tSNE graphs of concatenated data (4 animals per group) showing CD45+ gingival cells. **(E)** Comparison of the peak frequencies of PMN, T cells, and macrophages within CD45^+^ gingival cells over time. Dotted line represents day 3 (highlighting transition from innate to adaptive immune response) **(F)** Percentages and **(G)** cell number of neutrophils, T cells, and macrophages in gingiva. ^#^P<0.05 PMN *vs* T cell and Mac; ^&^P < 0.05 Mac *vs* T cell and PMN; *P < 0.05; **P 4< 0.01; ***P < 0.001.

To characterize the time-dependent frequency of the cellular components of the gingival inflammatory infiltrate within all CD45^+^ cells during the periodontal disease initiation and progression, we experimentally induced periodontitis in mice by placing ligatures for 1, 3, 5, or 10 days (**Figure 1A**). As previously observed by our group (22), the alveolar bone loss was significant by day five after the placement of ligatures (P<0.01) and reached its peak on day 10 (P<0.001; **Figures 1B, C**). To analyze the gingival immune infiltrate composition, we used the clustering algorithm FlowSOM and examined the behavior of 8 extracellular markers among the CD45^+^ gingival cells. We detected that at all time points, the three dominant populations were neutrophils (CD45^+^LY6G^high^LY6C^mid^CD11b^+/-^), macrophages (CD45^+^CD64^+^CD11b^+^MHCII^+^), and T cells (CD45^+^CD3^+^), which collectively represented >70% of all CD45^+^ cells (**Supplementary Figure 1**).

We then analyzed the shift in the frequency of these three cell populations as the disease progressed. We observed a stereotyped behavior (**Figures 1D, E**), where neutrophils peaked earlier than the other cell types, reaching their higher frequency 24h after the ligature placement (≈45%, P<0.05), suggesting an early activation of the innate immune response. Later (on day 3), macrophages reached their peak frequency (≈40%, P<0.05), indicating a transition to the adaptive immune response. T cells, which are the predominant immune cell type at the steady-state in the gingiva (P<0.05), showed a stable increase in their frequency, reaching similar levels as baseline by day 10 after the placement of ligatures (**Figures 1E, F**). When the cell numbers were analyzed, similar tendencies as frequencies were observed although the number of T cells decreased at later time points (**Figure 1G**).

### Early RvE1 Application Prevented the Progression of Bone Loss and Gingival T Cell Infiltration

**Figure 2:**
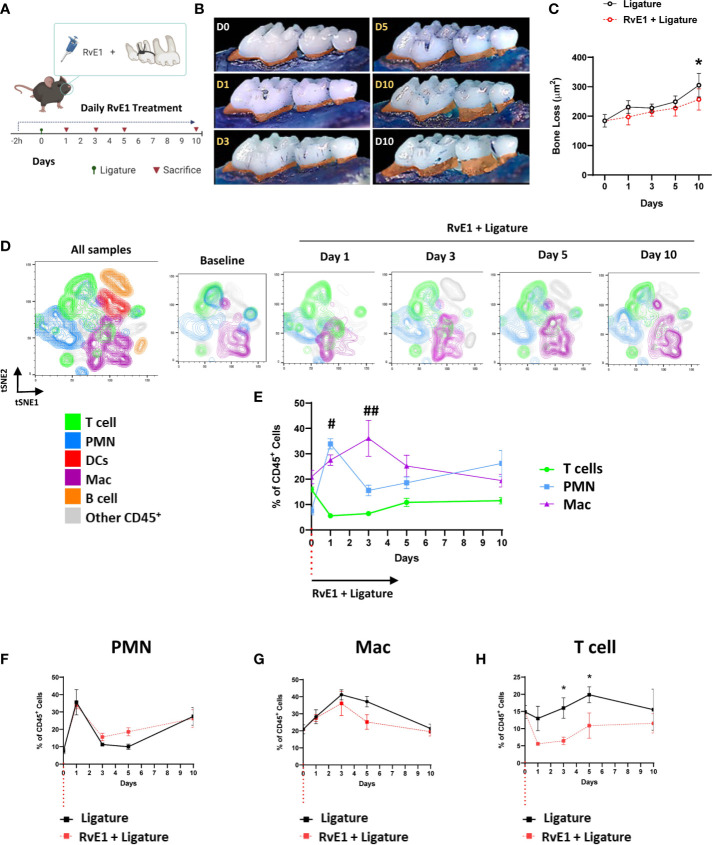
Temporal-dependent changes of the gingival immune-infiltrate in experimental periodontitis due to early RvE1 administration. **(A)** Experimental design. **(B)** Representative palatal views of maxillary molars from animals sacrificed at days 1, 3, 5, 10 after ligature application with or without RvE1 treatment (D1-D10 in yellow), baseline (D0) and ligature without treatment at day 10 (D10 white) as references. Bone loss area is highlighted in orange. **(C)** Bone loss in RvE1-treated and non-treated animals (n=5). **(D)** Identification of neutrophils (PMN, CD45^+^Ly6G^+^), macrophages (Mac, CD45^+^CD11b^+^CD64^+^), and T cell (CD45^+^CD3^+^) subsets in tSNE graphs of concatenated data (n = 4 animals per group) within CD45^+^ gingival cells. **(E)** Comparison of the frequencies of PMN, T cells, and macrophages within CD45^+^ gingival cells **(F**–**H)** Comparison of the frequency of each cell subtype within CD45^+^ gingival cells over time in animals with or without RvE1 treatment. ^#^P < 0.05 T cells *vs* PMN and Mac; ^##^P < 0.01 T cells *vs* PMN and Mac; *P < 0.05.

To assess the impact of RvE1 on the development of the gingival inflammatory infiltrate, we administered RvE1 topically daily, starting 2h before the placement of the ligatures (**Figure 2A**). The early administration of RvE1 had a clear impact on the prevention of the alveolar bone loss; the accumulated bone loss was significantly diminished compared to non-treated animals (P<0.05; **Figures 2B, C**). Daily administration of the vehicle we used to dilute the RvE1 (Sham) did not have any impact on the alveolar bone loss (**Supplementary Figure 2**).

To effectively monitor the changes in gingival immune composition, we then used a gating strategy that emulated the cell-population clustering identified initially by the FlowSOM algorithm (**Supplementary Figure 3A**). Using this gating strategy, we were able to identify the shift of the gingival immune cells and demonstrated the spatiotemporal changes in neutrophils, macrophages, and T cells as the three major cell populations at all time points (≈60%) (**Figures 2D, E**). We did not detect any significant differences between treated and untreated animals in the frequency of CD45^+^ cells among live cells in the gingiva (**Supplementary Figure 3B**). When we compared the kinetics of the frequencies of those three cell populations in non-RvE1-treated versus RvE1-treated animals, there were no significant changes in the innate immune cells (**Figures 2F, G**) while the gingival infiltration of T cells was significantly reduced on days 3 and 5 after the ligature placement in the RvE1 group (P<0.05; **Figure 2H**).

### Early RvE1 Application Reduced the Frequency of Effector T Cells in Cervical Lymph Nodes

**Figure 3:**
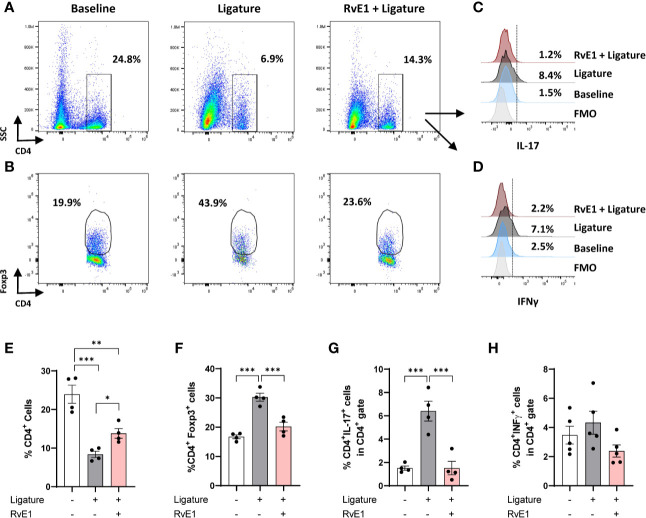
Impact of early RvE1 administration on CD4^+^ cells in cervical lymph nodes. **(A**, **B)** Representative dot plots indicating the frequency of CD4^+^ and CD4^+^Foxp3^+^ cells among all cells from cervical lymph nodes of animals with ligature-induced periodontitis (10 days; ligature), pre-treated with RvE1 (RvE1 + Ligature) or baseline (no ligature). **(C**, **D)** Representative histograms indicating the expression of IL-17 or IFNγ in CD4^+^ cells. **(E–H)** Frequency of total CD4+ cells, CD4^+^Foxp3^+^ cells, and CD4^+^ IL-17 or IFNγ^+^ cells in cervical lymph nodes (n = 4). FMO, Fluorescence minus one. *P < 0.05, **P < 0.01, ***P < 0.001.

To further corroborate the effect of RvE1 on the T-cell response, we analyzed the impact of early RvE1 application on CD4^+^ T cells in cervical lymph nodes at day 10 after the placement of ligatures (**Figure 3**). Animals with periodontitis showed a reduced frequency of CD4^+^ T cells within all cells in the lymph nodes as a direct result of increased organ size (lymphadenomegaly) compared to baseline (65% less, P<0.001) and RvE1-treated animals (42% less, P<0.01). RvE1-treated animals recovered the frequency of CD4^+^ cells significantly compared to non-treated animals (P<0.05; **Figures 3A, E**). The percentage of Foxp3^+^ cells in the CD4^+^ gate was increased in animals with periodontitis compared to both baseline and RvE1-treated animals (both ≈70% difference, P<0.001; **Figures 3B, F**). Finally, we analyzed the expression of IL-17 and IFNγ on CD4^+^ cells. We detected a significant reduction in the percentage of CD4^+^IL17^+^ cells in RvE1 group (≈75% less, P<0.001) comparable to healthy animals (**Figures 3C, G**). A similar tendency was observed with CD4^+^IFNy^+^ cells, although the differences were not significant (**Figures 3D, H**).

### Delayed RvE1 Application Selectively Inhibited the T-Cell Mediated Response

**Figure 4:**
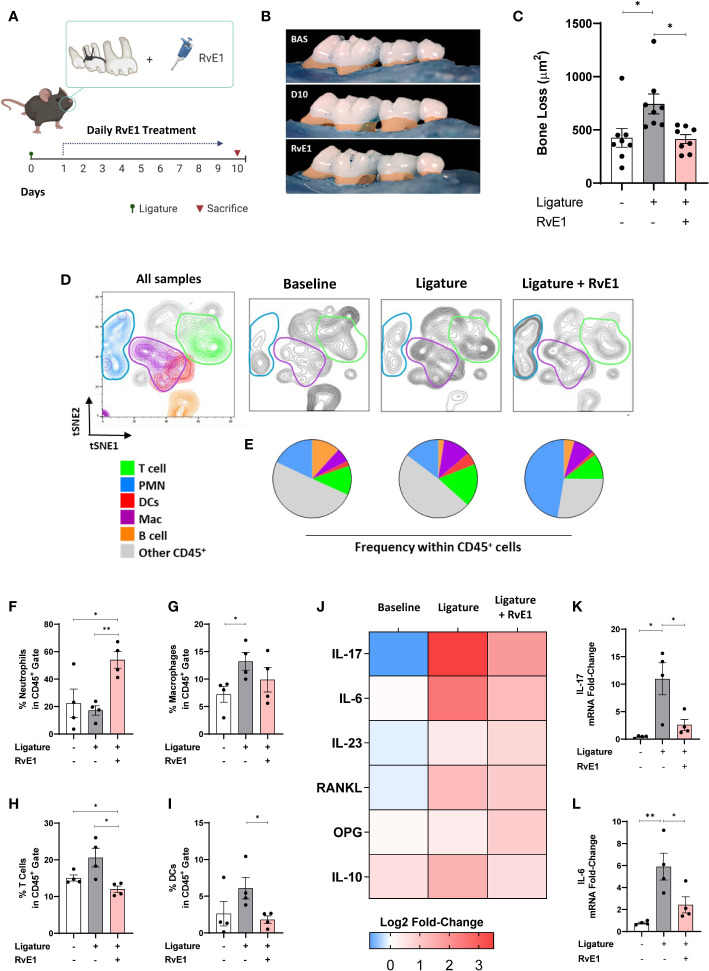
Impact of delayed RvE1 administration on the gingival immune-infiltrate composition. **(A)** Experimental design. **(B)** Representative palatal views of maxillary molars from animals at baseline (BAS), untreated ligature-induced periodontitis (D10), or treated with RvE1 after day 1 (RvE1). Bone loss area highlighted in orange. **(C)** Bone loss measurements from the cement-enamel junction to alveolar bone crest. **(D)** tSNE plots of concatenated data (n = 4 animals per group), and **(E)** pie charts indicating the frequency of neutrophils (PMN, CD45^+^Ly6G^+^), macrophages (Mac, CD45^+^CD11b^+^CD64^+^) and T cells (CD45^+^CD3^+^), Dendritic cells (DCs, CD45^+^CD11c^+^MHCII^+^), B cells (CD45^+^CD19^+^MHCII^+^), and other cells within gingival CD45^+^ cells. **(F–I)** Percentages of neutrophils, macrophages, T cells, and DCs in CD45^+^ gate. **(J)** Heat map representing the log2 fold-change gingival expression of mRNA of cytokines. **(K, L)** Gingival mRNA expression of IL-17 and IL-6. *P < 0.05, **P < 0.01.

Since RvE1 modulated neutrophil activity as an initial step in the induction of the inflammatory process, we used a delayed RvE1 administration to measure the T cell infiltration in response to RvE1 after the neutrophils were activated. The working hypothesis in this experiment was that T cell response could be directly impacted independent of neutrophil activation during the course of inflammation. We placed the ligatures for 24 hours, allowed the neutrophils to infiltrate the gingival tissues and then started the RvE1 application. We performed a single readout at day ten after the placement of ligatures (**Figure 4A**). The delayed administration of RvE1 significantly reduced alveolar bone loss by ≈40% (P<0.05; **Figures 4B,C**).

Using the same gating strategy as above, we compared the frequency of dominant cell populations in the CD45^+^ cells in gingival tissues (**Figures 4D, E**). T cell percentage was significantly reduced compared to non-RvE1-treated animals (P<0.05; **Figure 4F**). The percentage of DCs, which were the fourth dominant cell population among CD45^+^ cells in this particular experiment, were significantly reduced by the RvE1 administration (P<0.05; **Figure 4I**). Macrophages were not significantly impacted by the delayed application of the RvE1 (**Figure 4G**) while neutrophils were significantly increased in the RvE1 group compared to both untreated (P<0.05) and baseline animals (P<0.05), representing the predominant cell population (≈50% of all CD45^+^ cells, **Figure 4H**).

**Figure 5:**
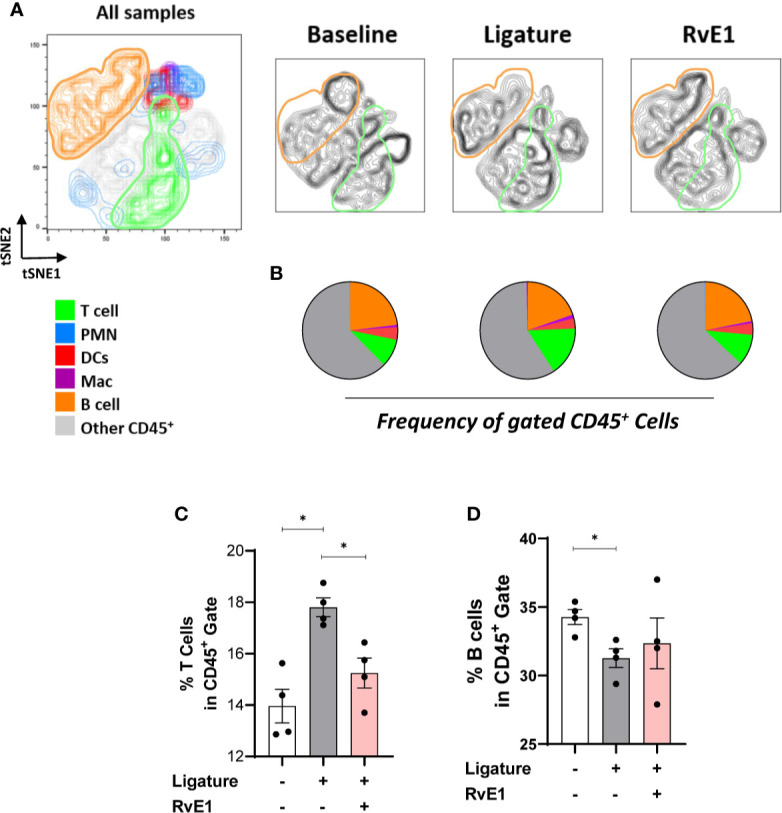
RvE1-induced changes in leukocyte composition in cervical lymph nodes. **(A)** tSNE plots of concatenated data (n = 4 animals per group), and **(B)** pie charts indicating the frequency neutrophils (PMN), macrophages (Mac), T cells, dendritic cells (DCs), B cells, and other cells within the CD45^+^ cell population in cervical lymph nodes from animals at baseline, untreated ligature-induced periodontitis (Ligature), or treated with RvE1 from day 1 (RvE1). **(C, D)** Percentages of T and B cells within CD45^+^ cells in cervical lymph nodes. *P < 0.05.

Next, we analyzed the mRNA expression of cytokines in the gingiva (**Figure 4J**). IL-17 and IL-6 were significantly reduced by the delayed RvE1 administration, reducing their expression in 78% and 65%, respectively, compared to non-treated animals (P<0.05; **Figures 4K, L**). In parallel, we measured the leukocyte composition in cervical lymph nodes (**Figures 5A, B**) and analyzed the percentage of T and B cells within CD45^+^ cells to identify their proliferative changes due to the inflammatory process. We detected a significant increase of T cell frequency in animals with periodontitis and a significant reduction in animals that received the delayed application of RvE1 (≈20% less, P<0.05; **Figure 5C**). There was no comparable impact on B cells (**Figure 5D**).

### Delayed RvE1 Application Restored Regulatory T Cell Response in Experimental Periodontitis

**Figure 6:**
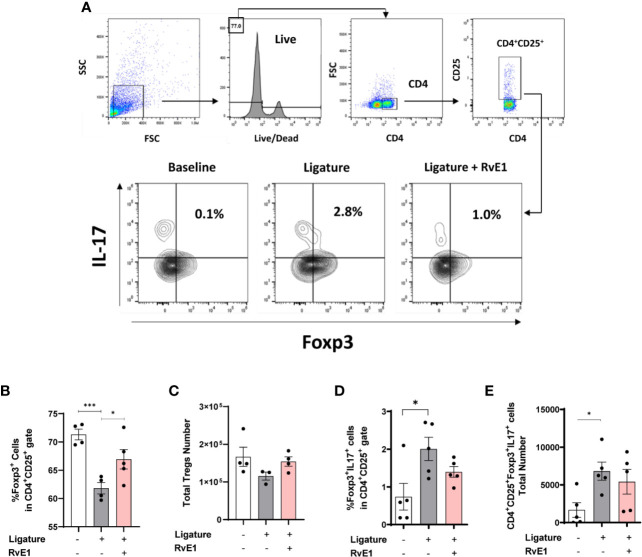
Impact of delayed RvE1 administration on Treg cells from cervical lymph nodes. **(A)** Representative plots indicating the gating strategy and percentage of CD4^+^CD25^+^Foxp3^+^IL17^+^ cells in cervical lymph nodes of animals at baseline, with ligature-induced periodontitis (Ligature), or treated with RvE1 from day 1 (RvE1). **(B)** Quantification of the percentage and **(C)** total number of Treg cells. **(D)** Quantification of the percentage and **(E)** cell number of IL-17^+^ Treg cells. *P < 0.05, ***P < 0.001.

Since the impact of RvE1 was profound on the T cell responses in both gingiva and cervical lymph nodes, we then tested whether this effect was primarily targeting the effector T cells, thus sparing regulatory T cells (Tregs; **Figure 6**). For this set of experiments, we used wild-type FVB mice. First, we examined the percentage of Foxp3^+^ cells among CD4^+^CD25^+^ cells. As expected, there was an imbalance between the proliferation of effector T cells (CD4^+^CD25^+^Foxp3^-^) and Tregs (CD4^+^CD25^+^Foxp3^+^) during the experimental periodontitis. Frequency of Tregs among CD4^+^CD25^+^ cells were reduced (P<0.001). RvE1 significantly recovered the Tregs frequency (P<0.05; **Figure 6B**). We also analyzed the expression of IL-17 on Tregs and detected a small but significant population of IL-17^+^Tregs in diseased animals (P<0.0.05), which was reduced in both percentage and number in the RvE1 group (**Figures 6A, D, E**).

## Discussion

In the present study, we measured the impact of RvE1 on the formation of the gingival inflammatory infiltrate and cell composition in cervical lymph nodes in a murine model of ligature-induced periodontitis. Using different experimental designs, we demonstrated that the RvE1 inhibited the infiltration of T cells in gingival tissues and effector T cell response in cervical lymph nodes. Our assessment of the immune cell populations infiltrating the gingiva at different time points indicated that neutrophils, macrophages, and T cells were the most prevalent leukocytes during the progression of periodontitis. The early administration of RvE1 dampened the gingival infiltration of T cells and reduced the frequency of CD4^+^IL-17^+^ T cells in lymph nodes without affecting neutrophils or macrophages. When we delayed the RvE1 administration to 24h after the placement of ligatures, we observed a reduced gingival T cell infiltration associated with less gingival IL-6 and IL-17 mRNA expression and dampened CD4^+^ proliferation in cervical lymph nodes. We also detected that RvE1 promoted the Treg response and prevented their phenotypic instability. Collectively, our data provides a new insight to the role of T cells during the progression of periodontitis and the impact of RvE1 administration on the periodontal immune response.

Our group has demonstrated that RvE1 prevents and regenerates the alveolar bone in various animal models of periodontitis (18–20). In these studies, end-point disease parameters were measured and kinetics of immune cell infiltrations were not assessed. Based on these studies, the mechanism of action of RvE1 seemed to be through neutrophils and macrophages and bone turnover was actively regulated by the restoration of osteoblastic/osteoclastic activities. Consistently, in a rat model of experimental periodontitis, RvE1 reduced the gingival expression of neutrophil chemoattractants such as CXCL1, CXCL2, and CCL3 (20). RvE1 effectively reduced the production of superoxide by neutrophils isolated from peripheral blood of patients with localized aggressive periodontitis, and increased the murine neutrophil phagocytosis of the periodontal pathogen *P. gingivalis* (18, 23). These functional changes were attributed to the binding of RvE1 to its cognate receptor, ERV1, expressed on neutrophils (23). RvE1 has also been shown to inhibit neutrophil infiltration in other inflammatory models, such as zymosan-induced peritonitis and dorsal air punch studies (21). Therefore, we expected to see less infiltration of neutrophils in animals with ligature-induced periodontitis that received early administration of RvE1. However, we did not detect such changes in the infiltration kinetics of neutrophils. Instead, we observed a significant alteration in the infiltration of T cells. CXCL1, CXCL2, and CCL3 production is dependent on the expression of Th17-related cytokines such as IL-17 and IL-23 (24, 25). Our research group previously demonstrated that at day five of experimental periodontitis begins the gingival over-expression of Th17-related cytokines, paired with increased differentiation of Th17 cells in cervical lymph nodes (22). Notably, the Th17 response commences while the alveolar bone loss becomes significant. Hence, we inferred that the reported reduced expression of IL-17-induced chemokines such as CXCL1, CXCL2, and CCL3 in experimental periodontitis was not necessarily associated with less neutrophil infiltration, but derived from a reduced Th17-response, which is associated with a decreased alveolar bone loss.

We detected a higher frequency of neutrophils among gingival CD45^+^ cells in animals with periodontitis that received a delayed administration of RvE1. Since we allowed the neutrophil response to occur normally, by postponing the treatment until after neutrophils peaked in frequency at the gingiva, our finding indicated that RvE1 promoted the renewal of early arrived gingival neutrophils at later stages of the periodontitis progression, and limited the T cell infiltration. Thus, RvE1 prolonged the innate response, which was associated with reduced alveolar bone loss. Neutrophils were once thought to be relatively monofunctional cells, with a role comprising purely the early recruitment to the site of injury to kill and remove infectious agents. However, the diversity of their immunomodulatory functions has become increasingly apparent (26), mainly through apoptosis, which provides a powerful anti-inflammatory signal. Neutrophils exert a sustained anti-inflammatory phenotypic reprogramming of macrophages, which is reflected by the sustained reduction in the release of pro- but not anti-inflammatory cytokines from macrophages (27). Recent evidence has demonstrated the suppressive role of neutrophils during microbial infections (28). For example, neutrophils are able to produce IL-10 following interaction with LPS-stimulated Tregs by direct cell-cell contact. IL-10^+^ neutrophils are also induced by exogenous IL-10 in a positive feedback loop. IL-10^+^ neutrophils have been detected in patients with periodontal abscess induced by Gram-negative bacteria (29). Since we detected a downregulated expression of the proinflammatory cytokines IL-17 and IL-6 in the periodontal tissues of animals treated with RvE1, it is possible to infer that the increased infiltration of neutrophils could be associated with an anti-inflammatory response, which in turn decreases the activation of the later adaptive immune response (26). However, further studies are needed to evaluate the direct impact of RvE1 on the resolution-phase associated functions of neutrophils, such as the production of IL-10 and their interaction with effector T cells and Tregs.

Emerging evidence indicates that SPMs regulate the adaptive immunity by modulating the development of B cells and T cells through direct contact or indirectly by changing the fate of antigen-presenting cells (30–32). RvE1 inhibits the inflammatory phenotype of different T cell-dependent pathologies. In an allergic airway inflammation model in mice, RvE1 reduced the expression of both IL-23 and IL-6, which are critical to sustaining the differentiation of Th17 cells (31). RvE1 inhibited the Th1/Th17 responses in corneal allograft transplantation in mice (33) and reduced the Th2-response in a murine model of asthma (34). Based on our previous description of the temporal progression of bone loss and appearance of Th17 markers in gingiva during ligature-induced periodontitis, we decided to analyze the changes in the gingival immune infiltrate due to RvE1 treatment for 10 days (22). We observed that both early and delayed administration of RvE1 altered the T cell response. Remarkably, we detected a decreased gingival frequency of T cells at day 3 and 5 after the ligature placement, a period that represents the transition between innate and adaptive immune responses. Our previous study demonstrated the significant lymphadenomegaly of cervical lymph nodes due to the periodontal inflammation in mice (22). Due to the significant increase in the total cell number of cervical lymph nodes, when we measured the frequency of CD4^+^ cells among all cell types (including non-leukocytes), the percentage decreased in animals with periodontitis compared to healthy mice. The opposite was observed when we analyzed the frequency of CD4^+^ cells among CD45^+^ cells, indicating CD4^+^ T cell proliferation during periodontitis. We also detected a significant reduction of CD4^+^IL-17^+^ cells in cervical lymph nodes, indicating a decreased Th17 response. Collectively, RvE1 rescued the periodontitis-induced changes in CD4^+^ cells. We recognize that the analysis performed is limited by the number of markers used simultaneously. A more detailed analysis is needed to characterize further which CD4^+^ T cell phenotypes and functions are being affected by the treatment as well as other less described immune compartments.

Tregs are essential modulators of the bone-destructive immune response and promoters of inflammatory resolution and tissue regeneration (35). We previously demonstrated that Treg phenotype and functions were altered during periodontitis, acquiring pro-inflammatory functions such as the expression of IL-17 (22). Since Treg stability depends on environmental cues that are significantly modified by the effector immune response-particularly Th17, we analyzed the impact of RvE1 on this paradigm (36). As documented before, when we analyzed the frequency of Foxp3^+^ cells among all CD4^+^ cells in cervical lymph nodes, the percentage increased in periodontitis as a feedback mechanism in the inflammatory process. On the contrary, the frequency of Foxp3^+^ cells within the CD4^+^CD25^+^ compartment, which only includes activated T cells, was reduced during periodontitis, suggesting an imbalance between effector and regulatory T responses (22). RvE1 recovered the frequencies of Tregs in both early and delayed application comparable to those levels in healthy animals. RvE1 reduced the small but significant population of IL-17^+^ Tregs detected in periodontitis. Hence, RvE1 restored the Treg/effector T cells balance and protected Treg regulatory phenotype.

Our results indicated that the RvE1 impacted the adaptive immune response selectively, since it regulates the effector but not the regulatory responses. A limitation of our study was that we did not identify whether RvE1 had a direct influence on T cells, by binding to its receptor, or an indirect impact through modulating other cell types that expressed cytokines and chemokines required for the differentiation and migration of effector T cells towards periodontal tissues. Given the amount of accumulated evidence indicating the robust effects of RvE1 on other cell types, we can infer that the outcome described on T cells is result of the net combination of functional cellular changes and the overall modulation of the inflammatory process; however, further research is needed to establish the direct role of RvE1 on CD4^+^ T cells differentiation and functionality.

Tipping the balance between regulatory and effector functions to restore a long-term ‘ceasefire’ is needed to reset a dysfunctional immune response therapeutically. The use of SPMs to promote the active resolution of inflammation constitutes an important approach that takes advantage of an endogenous mechanism with a wide range of biological actions. In this study, we identified the kinetics of major immune cell types in the gingival tissues. Our work has demonstrated T cells -particularly effector T cells- as the main impacted population by early and delayed administration of RvE1 during ligature-induced periodontitis independent of neutrophil involvement. These findings align with recent studies that highlighted the critical role for T cells in the inflammatory alveolar bone destruction providing new queries to further explore the role of RvE1 in the modulation of the adaptive immune response *in vivo*.

## Methods

### Animals

C57BL/6 and FVB wild type mice were purchased from the Jackson Laboratory (Maine, USA). 8-12-week-old male and female littermate animals were used. All animals were maintained in a pathogen-free environment, 24 ± 0.5°C, and 12:12 hours light/dark cycle at 40-70% of relative humidity. The experimental protocols were approved by the Institutional Animal Care and Use Committee of the Forsyth Institute.

### Ligature-Induced Periodontitis and RvE1 Administration

Experimental periodontitis was induced by placing 6.0-silk ligatures around the second bilateral upper jaws molars. Micro-Castroviejo forceps (Fine Science Tools), a magnification and cold-light source system, and an animal-holding structure were used to allow the maximum mouth opening of the anesthetized animal. Animals were subjected to ligatures at baseline (on day 0) of the experiment. In both early and delayed application of RvE1, the animals were treated daily with 10µL of 1µM RvE1 (Cayman chemicals) diluted in sterile phosphate-buffered saline (PBS) which was applied on the ligatures. To ensure the maximum bioactivity of RvE1, all aliquots were prepared for administration immediately before use in amber glass vials, replacing the inner vial air with nitrogen gas, and transported at 4°C.

### Bone Loss Measurements

Bilateral maxillary bones were defleshed by dermestids for 7 days at the animal facility at the Forsyth Institute. The bone samples were then cleaned of remaining debris, treated with 12% H_2_O_2_ for 4h, and stained with methylene blue. The images were taken at 0.63×10 magnification under a dissecting microscope (Axio observer A1, ZEISS) using the AxioVision 4.8 software. The area between the alveolar bone and cementoenamel junction (CEJ) on the palatal side of each maxillary molar was measured using the Fiji software (ImageJ) as bone loss.

### Flow Cytometry

Gingival samples from the mice were placed in RPMI-1640 + 1% penicillin-streptomycin (Sigma). The tissues were then treated with 0.15 µg/mL DNase I (GIBCO) + 3.2 mg/mL Collagenase Type IV (GIBCO) (Collagenase-DNase media) in 500 µL of RPMI-1640 + 1% penicillin-streptomycin in a 6-cm culture dish, as previously reported (37). The tissue sample minced with a sterile #15 scalpel blade. All tissue fragments were collected in a 15 ml tube containing 4 mL Collagenase-DNase media, and the tubes (one per animal) were placed in a water bath at 37°C for 1h. Fifty µL of 0.5M EDTA were added during the last 5 min of incubation. The samples were spun down at 1200 rpm for 10 min and the supernatant eliminated. The fragments were mashed onto a 70-µm cell strainer. The cells were filtered by washing the cell strainer repeatedly with RPMI-1640 + 1% penicillin-streptomycin + 0.15 µg/mL DNase I. The final cell suspension was spun down at 400*g* for 10 min; the supernatant was eliminated and the cells were resuspended in 200 µL of PBS +5% fetal-bovine serum (FBS). The cells were counted and stained using the LIVE/DEAD™ Fixable Yellow Stain kit (Invitrogen) according to the manufacturer’s instructions.

Then, the Fc receptors were blocked by TruStain FcX Antibody (Biolegend) according to the manufacturer’s instructions. The extracellular staining was performed in PBS containing 5% FBS, using the following antibodies for 30 min at 4°C in the dark: anti-I-A/I-E (M5/114.15.2, Biolegend), -CD3 (17A2, Biolegend), -CD19 (6D5, Biolegend), -CD64 (X54-5/7.1, Biolegend), -CD11b (M1/70, Biolegend), -CD11c (N418, Biolegend), -CD45 (30-F11, Biolegend), -Ly6G (IA8, Biolegend) and -Ly6C (HK14, Biolegend). We analyzed B lymphocytes (CD19^+^MHCII^+^), T lymphocytes (CD3^+^), macrophages (CD11b^+^ CD64^+^), dendritic cells (CD11c^+^ MHCII^+^), neutrophils (Ly6G^+^) and other cells (Ly6G^-^Ly6C^+/-^) within gate CD45^+^ (**Supplementary Figure 3**).

Single-cell suspensions were obtained from cervical lymph nodes using 70 µm cell strainers (Sigma-Aldrich) rinsed-out with PBS + 5% FBS. The extracellular staining was performed as done for gingival samples. For cytokine detection, 2-4×10^6^ cells were incubated for 4 hours in RPMI-1640 +10% FBS + 1% penicillin-streptomycin, 1/1000 Brefeldin A (eBioscience), 50 ng/ml PMA (Sigma), and 1 μg/ml Ionomycin (Sigma). Cells were then washed with PBS and stained for Live/Dead as explained before. The intracellular staining was done using a fixation/permeabilization staining kit following the manufacturer’s instructions (eBioscience) and using the following antibodies: anti-Foxp3 (MF-14, Biolegend), -CD4 (GK1.5, eBioscience), - CD25 (PC61, Biolegend) – IFNγ (XM61.2, Biolegend), and IL-17A (9B10, Biolegend).

All samples were analyzed on an Attune™ NxT acoustic focusing cytometer (Invitrogen). To set the flow cytometry compensation, the AbC™ Total Antibody Compensation Bead Kit (Thermo Fisher Scientific) was used according to the manufacturer’s instructions. The multiparametric data analysis was performed using the FlowJo software v10.7.1 (CA, USA). The data was analyzed using both a dimensionality reduction with the t-Distributed Stochastic Neighbor Embedding (tSNE) algorithm and the clustering FlowSOM algorithm. To effectively compare different samples and experiments, the following workflow was used: 1) Data clean up by applying manual gates to exclude doublets, debris, and dead cells from each sample, 2) Down sample of the CD45^+^ gated populations of each sample to 20,000 events, 3) Concatenate samples (4 samples per experimental group), 4) Dimensionally reduce (create tSNE parameters) on the concatenated file using default settings in FlowJo, iterations 1000, perplexity 30, and learning rate (eta) 521, and 5) Analysis of every population and identifying phenotypes using FlowSOM and a later established gating strategy (**Supplementary Figures 1**, **3**).

### Gingival RNA Extraction and qRT-PCR

The gingival collar around all maxillary molars, without including palatal or buccal mucosa, was carefully excised using scalpel blade (#15) under a dissecting microscope. All samples were transported in RNAlater (Life Technologies). To lyse the samples, we used glass tissue homogenizers and 300 µL of RLT lysis buffer (Qiagen), then we further homogenized the samples in QIAshredder columns (Qiagen). The RNA was obtained from the homogenate using the RNeasy Mini Kit (Qiagen), following the manufacturer’s instructions. The first-strand of cDNA was synthesized from 1 µg of RNA using the reverse transcription kit (SuperScript III, Invitrogen), according to the manufacturer’s instructions. Quantitative real-time PCR amplification of cDNAs (50 ng) was performed using TaqMan™ Fast Advanced Master Mix (Thermo Fisher) and the respective TaqMan probe for each targeted gene. The Step One™ Real-Time PCR System (Applied Biosystems) instrument was used for quantification. All transcript levels were normalized to transcript levels of mouse18S rRNA. The data was presented as a fold-change of relative quantity using the 2^−ΔΔCt^ method.

### Statistical Analysis

All data sets were analyzed using Prism 8 software v8.4.2 (GraphPad). The data distribution was analyzed using the Shapiro-Wilk test. To determine differences between two experimental groups, unpaired Student-t or Mann-Whitney-U tests were performed. ANOVA or Kruskal-Wallis tests were used when more than two groups were compared, followed by multiple comparison Tukey or Dunn post-hoc tests. P values < 0.05 were considered statistically significant.

### Original Figures

Created with BioRender.com.

## Data Availability Statement

The original contributions presented in the study are included in the article/**Supplementary Material**. Further inquiries can be directed to the corresponding author.

## Ethics Statement

The animal study was reviewed and approved by the Institutional Animal Care and Use Committee of the Forsyth Institute.

## Author Contributions

CA designed the study, carried out the experiments, analyzed the data, and drafted the manuscript. SS and HA contributed to the experiment’s readout. PR analyzed bone loss. Y-CW contributed to the flow cytometry data analysis. RA contributed to the maintenance of the animal colonies and animal treatments. MG, RV, and AK conceived the study and helped to draft the manuscript. All authors contributed to the article and approved the submitted version.

## Funding

This research was supported by grants from the NIH/NIA R01AG062496. CA is an awardee of the fellowship CONICYT 21161255 from the Chilean Government.

## Conflict of Interest

The authors declare that the research was conducted in the absence of any commercial or financial relationships that could be construed as a potential conflict of interest.
