# Corrigendum: RvE1 Impacts the Gingival Inflammatory Infiltrate by Inhibiting the T Cell Response in Experimental Periodontitis

**DOI:** 10.3389/fimmu.2022.936102

**Published:** 2022-05-19

**Authors:** Carla Alvarez, Henrique Abdalla, Salwa Suliman, Paola Rojas, Yu-Chiao Wu, Rawan Almarhoumi, Ren-Yeong Huang, Mario Galindo, Rolando Vernal, Alpdogan Kantarci

**Affiliations:** ^1^ Forsyth Institute, Cambridge, MA, United States; ^2^ Laboratory of Neuroimmune Interface of Pain Research, Faculdade São Leopoldo Mandic, Instituto de Pesquisas São Leopoldo Mandic, Campinas, Brazil; ^3^ Department of Clinical Dentistry, Center for Clinical Dental Research, University of Bergen, Bergen, Norway; ^4^ Harvard School of Dental Medicine, Boston, MA, United States; ^5^ School of Dentistry, National Defense Medical Center, Taipei, Taiwan; ^6^ Program of Cellular and Molecular Biology, Institute of Biomedical Sciences (ICBM), Faculty of Medicine, Universidad de Chile, Santiago, Chile; ^7^ Millennium Institute on Immunology and Immunotherapy, Faculty of Medicine, University of Chile, Santiago, Chile; ^8^ Periodontal Biology Laboratory, Dentistry Faculty, Universidad de Chile, Santiago, Chile

**Keywords:** T cells, Periodontal disease, inflammation, RvE1, SPMs (specialized pro-resolving mediators)

An author name was incorrectly spelled as **Salwa Sulliman**. The correct spelling is **Salwa Suliman**.

The authors state that this does not change the scientific conclusions of the article in any way. The original article has been updated.

## Publisher’s Note

All claims expressed in this article are solely those of the authors and do not necessarily represent those of their affiliated organizations, or those of the publisher, the editors and the reviewers. Any product that may be evaluated in this article, or claim that may be made by its manufacturer, is not guaranteed or endorsed by the publisher.

